# Is (*S*)-norketamine an alternative antidepressant for esketamine?

**DOI:** 10.1007/s00406-018-0922-2

**Published:** 2018-07-14

**Authors:** Kenji Hashimoto, Chun Yang

**Affiliations:** 1 Division of Clinical Neuroscience, Chiba University Center for Forensic Mental Health, 1-8-1 Inohana, Chiba, 260-8670 Japan; 2 Department of Anesthesiology, Tongji Hospital, Tongji Medical College, Huazhong University of Science and Technology, Wuhan 430030, China

**Fig. 1:**
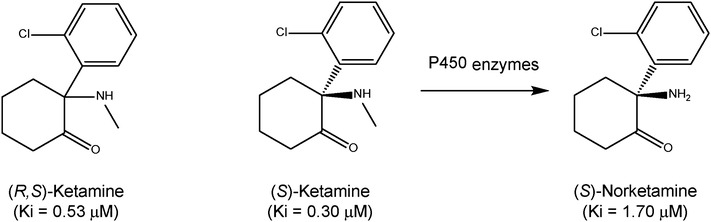
Chemical structure of (*R,S*)-ketamine, (*S*)-ketamine (or esketamine) and its metabolite (*S*)-norketamine. (*S*)-ketamine is metabolized into (*S*)-norketamine by the microsomal cytochrome P450 system. The values in parentheses are the inhibitor constant values for NMDAR (10)

The *N*-methyl-d-aspartate receptor (NMDAR) antagonist (*R,S*)-ketamine has been hailed as the most important advance in the treatment of depression for the past 50 years. The rapid and sustained antidepressant effects of (*R,S*)-ketamine have spurred a great deal of research interest, with growing off-label use for the treatment of depression, although the concerns about the safety of repeated (*R,S*)-ketamine infusions persist [1].

(*R,S*)-ketamine (Ki = 0.53 µM for NMDAR) is a racemic mixture containing equal parts of (*R*)-ketamine (or arketamine) (Ki = 1.4 µM for NMDAR) and (*S*)-ketamine (or esketamine) (Ki = 0.30 µM for NMDAR) (Fig. 1). Previously, we reported that (*R*)-ketamine showed greater potency and longer lasting antidepressant effects than esketamine in animal models of depression [2]. Unlike esketamine, (*R*)-ketamine does not induce psychotomimetic side effects or exhibit abuse potential in rodents [3]. A positron emission tomography study using conscious monkeys demonstrated a marked reduction in dopamine D_2/3_ receptor binding in the monkey striatum after a single infusion of esketamine (0.5 mg/kg for 40 min), suggesting a marked release of dopamine from presynaptic terminal [4]. Interestingly, it is reported that a single infusion of esketamine (0.5 mg/kg for 40 min) caused psychotomimetic and dissociative symptoms (e.g., strange experiences like a feeling of floating in outer space or depersonalization/derealization) in patients with treatment-resistant depression [5]. Therefore, it is suggested that esketamine-induced dopamine release might be associated with the acute psychotomimetic and dissociative side effects in humans. Collectively, (*R*)-ketamine could be a safer antidepressant in humans than esketamine [2–4] .

The Janssen Pharmaceutical Companies of Johnson & Johnson has been developing esketamine nasal spray as the novel antidepressant. Two phase 2 randomized clinical trials of intranasal esketamine have been reported. Daly et al. [6] reported the efficacy of intranasal esketamine (28, 56, or 84 mg twice weekly) for the rapid reduction of symptoms of depression and suicidality in treatment-resistant patients. Change in Montgomery–Åsberg Depression Rating Scale (MADRS) total score in all three esketamine groups was superior to placebo, with a significant dose–response relationship [6]. Furthermore, Canuso et al. [7] reported the efficacy and safety of intranasal esketamine for the rapid reduction of symptoms of depression and suicidality in patients at imminent risk for suicide. Esketamine (84 mg twice weekly for 4 weeks) along with antidepressant treatment significantly decreased suicidal symptoms and depression at both 4 and 24 h; however, these effects did not differ from those of placebo after 4 weeks of treatment. In contrast, differences in suicide risk scores, as determined by clinical global judgment, were not statistically significant between the groups at any time point [7]. Dissociative symptoms began shortly after each infusion and attenuated after repeated doses. The abuse potential of intranasal esketamine was not specifically examined within this short trial [6, 7]. It seems that the effects of intranasal esketamine may be less potent than intravenous (*R,S*)-ketamine. At the American Psychiatric Association annual meeting in May 2018, the company presented mixed findings of two phase 3 trials for intranasal esketamine [8]. Adult treatment-resistant patients who received esketamine had a significantly greater drop in MADRS score from baseline at day 28 compared with placebo group. In contrast, 65 and older patients with treatment-resistant depression who received esketamine plus a newly initiated oral antidepressant had no statistical difference in change in the MADRS score from baseline to day 28 compared with placebo group [8].

The potential for ketamine abuse is one of the most important drawbacks of repeated ketamine infusions for treating mood disorders [9]. Ketamine-induced rewarding effects are associated with the potent inhibition of the NMDAR in the brain. Esketamine is metabolized into (*S*)-norketamine (*K*_*i*_ = 1.70 µM for NMDAR) by the microsomal cytochrome P450 system (Fig. 1). Preclinical studies have revealed the abuse liability of esketamine in rodents, although its metabolite (*S*)-norketamine does not possess abuse liability in rodent model [10]. Given the role that NMDAR inhibition plays in the abuse potential of ketamine, clinicians should carefully monitor signs of abuse potential for (*R,S*)-ketamine and esketamine in their patients.

Very recently, we reported that (*S*)-norketamine exhibits rapid and sustained antidepressant effects in rodent models of depression, with a potency similar to that of the antidepressant actions of esketamine [10]. (*S*)-norketamine’s antidepressant effects are less potent than (*R*)-ketamine. Unlike esketamine, (*S*)-norketamine does not cause behavioral and biochemical abnormalities, such as prepulse inhibition deficits, abuse potential, loss of parvalbumin immunoreactivity in the medial prefrontal cortex, or increases in baseline γ-oscillations [10]. Given the lower affinity of (*S*)-norketamine for NMDAR, repeated infusions of (*S*)-norketamine may have fewer detrimental side effects in humans than its parent compound esketamine; however, additional long-term studies of repeated infusions of these compounds are warranted.

In conclusion, (*S*)-norketamine could be a safer alternative antidepressant than esketamine for use in humans.
